# Protease shaving of *Mycobacterium tuberculosis* facilitates vaccine antigen discovery and delivery of novel cargoes to the Mtb surface

**DOI:** 10.1128/spectrum.02277-24

**Published:** 2024-12-17

**Authors:** Bianca A. Lepe, Christine R. Zheng, Owen K. Leddy, Benjamin L. Allsup, Sydney L. Solomon, Bryan D. Bryson

**Affiliations:** 1 Department of Biological Engineering, MIT, Cambridge, Massachusetts, USA; 2 Ragon Institute of Mass General, Harvard, and MIT, Cambridge, Massachusetts, USA; 3 Koch Institute for Integrative Cancer Research, MIT, Cambridge, Massachusetts, USA; University of Hawaii at Manoa, Honolulu, Hawaii, USA

**Keywords:** tuberculosis, cell surface, proteomics, surface antigens

## Abstract

Tuberculosis (TB) is the leading cause of infectious disease death and lacks a vaccine capable of protecting adults from pulmonary TB. The bacterial surface is a critical interface that shapes host-pathogen interactions. Several knowledge gaps persist in our understanding of *Mycobacterium tuberculosis* (Mtb)-host interactions that may be addressed by an improved understanding of the Mtb surface proteome, including the identification of novel vaccine targets as well as developing new approaches to interrogate host-pathogen interactions. Here, we sought to expand our understanding of the Mtb surface proteome in service of these knowledge gaps by adapting protease shaving protocols for multiplexed quantitative mass spectrometry. Pairing quantitative mass spectrometry with the construction of validation strains, we revealed several novel Mtb proteins on the Mtb surface largely derived from the PE/PPE class of Mtb proteins, including PPE18, a component of a leading Mtb vaccine candidate. We next exploited the localization of PPE18 to decorate the Mtb surface with heterologous proteins. Together, these studies reveal potential novel targets for new Mtb vaccines as well as facilitate new approaches to study difficult-to-study cellular compartments during bacterial growth and infection.

**IMPORTANCE:**

The surface of a bacterial pathogen is a critical interface between the bacterium and the immune system. A better understanding of this interface would facilitate the discovery of new vaccine targets, new virulence proteins, and enable new technologies that modify the bacterial surface. In this study, we established a multiplexed and quantitative biochemical strategy to study the surface of *Mycobacterium tuberculosis* (Mtb) and identified new vaccine targets. We furthermore established design rules for new technologies aimed at modifying the composition of the bacterial surface. Specifically, we achieved a biological milestone that has not been rigorously reported previously, which is the successful modification of the Mtb surface with a non-native protein.

## INTRODUCTION

Tuberculosis (TB), caused by *Mycobacterium tuberculosis* (Mtb), is the leading cause of death due to a single infectious agent (1). No protective vaccine has been developed that meets the target product profile needed in humans to alter the course of the TB pandemic. Gaps in our understanding of the host-Mtb interactions that facilitate interactions between the host immune system and the bacterium limit the development of more effective TB vaccines, host-directed therapies, and biotechnologies suited to generate new insights about Mtb infection.

Interactions between Mtb proteins and the host underlie both innate and adaptive immune responses. For example, secreted Mtb proteins play a critical role in immune responses. The ESX-1 and 3 secretion systems have been shown to secrete proteins that interact with the endolysosomal system in phagocytes modulating both membrane damage and repair pathways (2–7). In addition to modulating innate immune processes, secreted Mtb proteins comprise a significant fraction of the antigenic targets of Mtb-specific T cells that facilitate immune protection during infection (8, 9). These data emphasize the critical role that Mtb proteins, defined by their physical localization in the bacterium, influence immune responses. Technically, the identification of secreted Mtb proteins has been greatly facilitated by optimized biochemical workflows that enable the physical separation of the Mtb secretome from other proteomic fractions (10–13).

Recent studies also emphasize that Mtb proteins present on the mycobacterial surface may serve a similar role in bacterial pathogenesis and interactions with adaptive immunity. PPE51, an Mtb porin, was recently demonstrated to be present on the mycobacterial surface and facilitate nutrient acquisition, a critical function required for bacterial survival (14). We and others have also demonstrated that beyond its nutrient acquisition function, PPE51 serves as an antigenic target for CD8+ T cells (15, 16). Similar observations have been made for other pathogens. In Gram-negative bacteria, there is a set of highly conserved surface porins that play a role in small molecule transport across the membrane and bacterial homeostasis. For example, porin proteins OmpC and OmpF in *Salmonella enterica* serovar Typhi both induce a robust cell-mediated immune response in mice and humans (17).

Despite these examples emphasizing a role for surface proteins in Mtb pathogenesis, considerably less is known about the composition of this compartment compared to the Mtb secretome (18). Physical fractionation of the different Mtb compartments has been performed; however, these fractionation methods are equipment intensive due to the requirement of ultracentrifugation, which is difficult to perform in a BSL3 environment. Additionally, ultracentrifugation methods alone are insufficient to define the topology of proteins in the mycobacterial surface. Addressing these gaps in our understanding of the surface proteome would facilitate an improved understanding of Mtb biology as well as potentially facilitate new approaches to characterize the host-pathogen interface. For instance, Olson and colleagues (19) previously demonstrated in *Chlamydia trachomatis* that using proximity labeling on proteins at the host-pathogen interface captures novel *in vivo* protein-protein interactions during development, which enables a new modality for understanding how chlamydiae survive in their intracellular niche.

We drew inspiration from previous studies where the surface proteome of bacteria was defined by transiently adding proteases to axenic cultures of live bacteria to liberate peptides derived from surface-accessible proteins (20, 21). We hypothesized that these methods could be adapted for virulent Mtb to define the surface proteome and improved upon by (i) using an existing known Mtb surface protein to optimize proteolysis conditions, (ii) application of isobaric mass tag-based quantification, and (iii) orthogonal validation of surface localization using flow cytometry. We applied these improvements to the Mtb strain, H37Rv, under BSL3 conditions. Our studies expanded the number of PE and PPE proteins validated to be present at the Mtb cell surface, established design rules for fusions to these surface proteins, and established a generalizable strategy to install heterologous cargo on the Mtb surface using these novel proteins as scaffolds.

## RESULTS

### Protease shaving can be applied for the study of virulent Mtb

**Fig 1:**
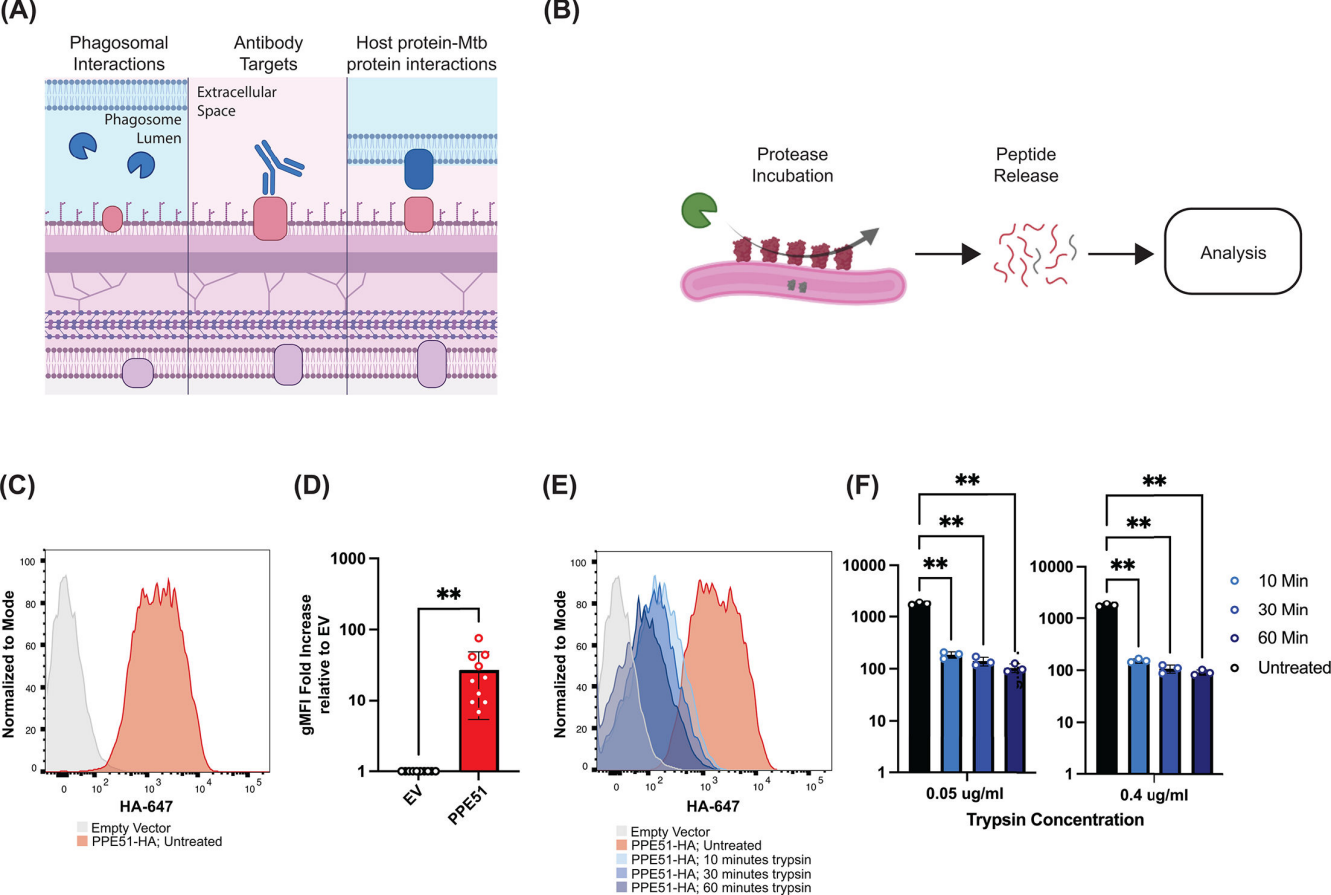
Protease shaving enables the identification of Mtb surface proteins. (**A**) Schematic of the Mtb surface proteome and potential points of interaction between the Mtb surface proteome and the host immune response. (**B**) Protease shaving methodology. Intact live Mtb are exposed to protease for defined periods of time, and peptides liberated into the supernatant are identified and/or quantified by mass spectrometry. (**C**) Flow cytometry analysis of HA surface expression on an H37Rv strain expressing an empty vector PPE51-HA. Representative of 10 independent biological replicates. (**D**) Quantification of HA surface expression on empty vector or PPE51-HA H37Rv strain (**P < 0.01, paired *t*-test, *n =* 10)*.* (**E**) Flow cytometry analysis reveals loss of PPE51-HA signal following trypsin (0.4 µg/mL) exposure. Representative of three independent replicates. (**F**) Quantification of HA surface expression on PPE51-HA expressing Mtb over time at two different trypsin concentrations (0.4 and 0.05 µg/mL) (**P < 0.01; two-way ANOVA with Tukey’s multiple comparisons test, *n =* 3 or 4 independent biological replicates).

A major gap in our understanding of Mtb is the localization of Mtb proteins, especially of those proteins that are accessible on the Mtb surface. Addressing this gap in knowledge would facilitate the rational design of antibodies that can target intact Mtb or better identify Mtb proteins that can interact with host machinery during the earliest host-pathogen interactions (Fig. 1A). Previous studies have successfully identified and validated Mtb surface proteins. Wang and colleagues (14) leveraged bacterial genetics to recover a PPE51 mutant that was resistant to 3bMP1, a novel inhibitor of Mtb growth and survival. Subsequent biochemical analysis using an epitope-tagged PPE51 combined with ultracentrifugation or flow cytometry revealed that PPE51 was accessible on the Mtb surface. Previous reports suggest that CpnT is localized to the Mtb surface; however, our efforts to reproduce these data did not show convincing surface localization (data not shown) (22). In a study of genes required for heme utilization, Mitra and colleagues (23) identified PPE36 and PPE62 as surface PPE proteins. In *Mycobacterium marinum,* Savijoki and colleagues (21) utilized a protease shaving protocol to identify proteins on the surface of planktonic *M. marinum*; however, the authors did not orthogonally demonstrate the localization of the proteins they implicated as being on the surface.

We drew inspiration from the prior work cited above in addition to the work in *Streptococcus pyogenes* to define the surface proteome of Mtb. In protease shaving, intact and live bacteria are transiently incubated with a protease to liberate protease-accessible peptides from the bacterial surface (Fig. 1B) (20, 21). Liberated peptides are then identified using liquid chromatography and tandem mass spectrometry.

We first sought to test the feasibility of utilizing protease shaving to determine the Mtb surface proteome. To establish feasibility as well as optimize our protocol, we leveraged the previously identified Mtb surface protein, PPE51, to define the biochemical parameters that govern successful protease shaving in Mtb. We generated an H37Rv strain expressing PPE51 with a C-terminal HA tag. Liberation of the HA tag from PPE51 would represent the successful shaving of peptides from the Mtb surface and can be easily read out via flow cytometry. We first utilized indirect flow cytometry using an anti-HA antibody to validate that PPE51 was antibody accessible without fixation or permeabilization of the bacteria. Compared to an empty vector, the antibody signal was significantly higher in Mtb expressing PPE51-HA (Fig. 1C and D, representative of 10 independent biological replicates). After confirming that the HA tag on PPE51 was surface accessible, we next sought to identify protease incubation times and concentrations that resulted in the loss of HA staining on the Mtb surface. We selected trypsin as our enzyme of choice for surface shaving due to the vast amounts of publicly available mass spectrometry data of Mtb compartments analyzed following trypsin digestion, which could serve as appropriate data sets for comparison. Other proteases with alternative cleavage preferences could be used in this assay with slight modifications. We incubated Mtb in a sucrose buffer with two different concentrations of trypsin (0.4 and 0.05 µg/mL, see Materials and Methods) and across three different incubation times (10, 30, and 60 minutes). For both trypsin concentrations, we saw a reduction in HA signal as early as 10 minutes. At all time points, both protease concentrations resulted in a statistically significant decrease in HA signal as quantified by flow cytometry (Fig. 1E and F, *n* = 3 biological replicates). We next sought to confirm that our trypsin shaving experiments would result in peptides detectable by mass spectrometry, so we generated mass spectrometry data to confirm this was the case. Using unlabeled, discovery mass spectrometry, we identified >1,300 proteins with at least two peptides per protein, including PPE51 (Table S1). Together, these results suggested that exposure to trypsin can digest Mtb surface proteins and cleave Mtb-derived peptides, thus confirming the utility of protease shaving to define the surface proteome of Mtb.

### Quantitative protease shaving reveals the surface proteome of Mtb

Our flow cytometry data show that protease shaving liberates Mtb peptides from the cell surface, and our preliminary mass spectrometry data revealed that this protocol would result in Mtb peptides detectable by mass spectrometry. Given this success, we next sought to use this technique to identify novel proteins in the Mtb surface proteome. Based on our results with PPE51, we decided to perform all experiments with a 30-minute incubation with 0.4 µg/mL trypsin. We initially compared the proteins we identified in our pilot surface shaving mass spectrometry experiments with additional mass spectrometry data sets of other compartments. We found many proteins that overlapped between the pilot surface shaving data and published data sets of other Mtb compartments. We reasoned that autolysis of Mtb might negatively impact our ability to identify *bona fide* Mtb surface proteins, so we devised a quantitative workflow to distinguish between autolysis and Mtb surface proteins.

Our approach is as follows: Phthiocerol dimycocerosate (PDIM)-positive Mtb was grown to the late log phase in 7H9 supplemented with oleic acid-albumin-dextrose-catalase (OADC) and tyloxapol. Mtb was then washed in sucrose buffer and split into two identical tubes. In one tube, we added trypsin (0.4 µg/mL) to sucrose buffer containing Mtb and incubated Mtb for 30 minutes prior to separating Mtb from the supernatant. We reasoned that it was possible that Mtb autolysis could occur during this same time frame, which would imply that our cell-free supernatant from the sample incubated with trypsin would include both digested surface proteins as well as digested proteins derived from autolysis. We thus used the second identically prepared tube and incubated it identically to the first sample except intact Mtb was not exposed to trypsin directly. We reasoned that in this second sample, only proteins derived from autolysis would be in the supernatant. After 30 minutes, we collected this supernatant which ostensibly contains proteins from autolysis and then subjected it to a tryptic digestion. We reasoned that a quantitative comparison between these two conditions would aid in distinguishing between Mtb proteins from autolysis and Mtb surface proteins.

Given that mass spectrometry performed with data-dependent acquisition settings is not guaranteed to identify the same peptide ions across multiple experiments of the same sample type, we chose to multiplex our experiments using isobaric mass tags to label multiple biological replicates. Three independent biological replicates of the experiment described above were pooled into a single quantitative mass spectrometry analysis to maximize peptide identification and overlap.

**Fig 2:**
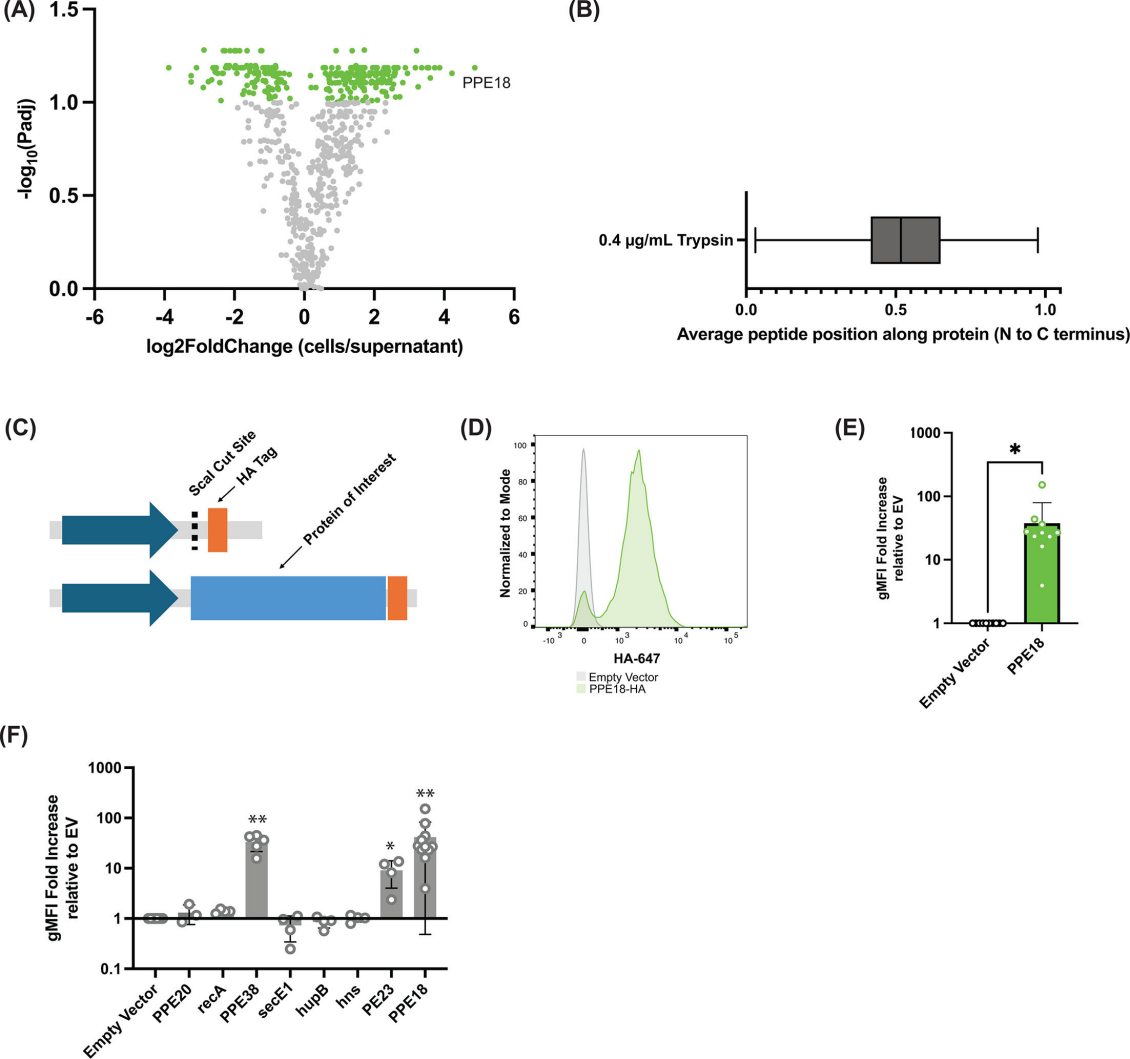
Quantitative mass spectrometry analysis of protease shaving experiments and validation of results. (**A**) Volcano plot of protease shaving results. The log FC indicates the abundance ratio of all proteins identified (trypsinized Mtb/trypsinized autolysis control). Each point represents one protein. Light green dots indicate proteins with a positive fold change greater than 2, with an adjusted *P*-value of 0.1 or less. (**B**) Box and whiskers plot of the average peptide position of protease shaving results. (**C**) Plasmid design of surface display vector for the validation of protease shaving mass spectrometry results. Each Mtb protein was cloned downstream of a GroEL2 promoter and upstream of a glycine-serine linker and HA tag. (**D**) Flow cytometry analysis of PPE18-HA compared to an empty vector (representative of 10 independent experiments). (**E**) Quantification of geometric mean fluorescence intensity of HA signal across PPE18-HA and empty vector Mtb strains (*P < 0.05, paired *t*-test, *n =* 10). (**F**) Summary of results across all validation strains constructed. (*P < 0.05 and **P < 0.01; paired *t*-test between validation strain and empty vector).

Our mass spectrometry experiments identified 694 Mtb proteins. We identified 167 proteins with an elevated abundance (greater than twofold) in the samples derived from trypsin incubation with Mtb compared to the autolysis control supernatant (Fig. 2A; Table S2). The 167 proteins with a fold change > 2 in the intact Mtb condition included a variety of protein types, including SecE, a component of the Sec secretion machine, PPE18, a subunit vaccine antigen, and LucA, a protein involved in Mtb lipid import. One of our top hits, PPE38, was previously shown to be exposed on the surface of *Mycobacterium marinum,* suggesting potentially conserved protein localization features across mycobacteria (24, 25). Furthermore, we also recovered PPE51, which we had used in our optimization experiments. The proteins with a fold change greater than 2 compared to the autolysis control in our mass spectrometry data ranged in size from ~8 to 300 kDa.

Compartment-specific mass spectrometry studies in Mtb often just report the proteins identified by mass spectrometry. We sought to orthogonally validate our mass spectrometry data. Since we wanted to test multiple different proteins and given the paucity of monoclonal antibodies available for Mtb proteins, especially those we identified, we opted for a generalizable alternative strategy that did not rely on raising antibodies for each protein identified. We drew inspiration from prior studies that demonstrated surface localization using indirect flow cytometry of Mtb protein fusions with small epitope tags (14, 23). We selected the top 10 proteins based on fold change from our quantitative mass spectrometry experiments for targeted validation by strain construction and flow cytometry.

We first examined the peptides detected by mass spectrometry from the top N proteins we selected and conducted an N- vs C-terminal bias analysis. Specifically, we sought to determine if there was a positional bias to the peptides detected in our experiments relative to the source protein to justify our epitope tagging strategy. We reasoned that quantifying this positional bias would facilitate a generalizable cloning strategy where a common vector backbone could be designed, including an epitope tag that the candidate surface protein could subsequently be subcloned into. For each Mtb protein with a twofold change in the trypsin-containing condition and an adjusted *P*-value < 0.1, we quantified the average relative position of the identified peptides along the protein from whence it came. Proteins with peptides largely originating from the N-terminus of the protein would have an average relative position value closer to 0, whereas peptides originating from the C-terminus would have a value closer to 1. The average relative peptide position was 0.53, which did not clearly indicate whether N- or C-terminal tagging would be ideal. Therefore, we opted for a C-terminal tagging strategy based on prior work tagging Mtb surface proteins (Fig. 2B) (14, 23). We generated a Gibson Assembly-compatible episomal vector consisting of a strong promoter (*GroEL2*) followed by a ScaI cut site, a short linker (GGSGSS, 0.45 kDa), and an HA tag (1.1 kDa) (Fig. 2C). After generating the plasmids for the validation strains, we transformed them all into H37Rv. Of the 10 plasmids transformed, we successfully recovered colonies from eight transformations.

Recovered Mtb transformants were grown to the late log phase and then stained for the HA epitope tag using indirect flow cytometry. In each experiment, an empty vector control was stained as a negative control. We first tested the Mtb strain expressing PPE18-HA and compared it to an empty vector strain and found that the HA signal was much higher in the PPE18-HA strain compared to the empty vector strain (Fig. 2D and E, *n* = 10 independent biological replicates). For each strain, the geometric mean fluorescence intensity (gMFI) of the antibody signal was calculated and then normalized relative to the empty vector for at least three independent biological replicates. Of these eight strains, three of them showed a statistically significant difference in gMFI compared to the empty vector. These proteins included PPE18, PPE38, and PE23 (Fig. 2D through F). Notably, all these proteins are PE/PPE proteins, which is consistent with other targeted studies in the field showing these proteins on the Mtb surface. Proteins that were not validated by C-terminal tagging and flow cytometry included hup, secE, and PPE20. We carefully inspected these proteins that did not validate based on these results for any potential biochemical insight that might explain their failed validation in this assay. In the mass spectrometry data, there was no appreciable difference in the percentage of the protein identified or the number of unique peptides from the source protein (Fig. S1).

We next sought to confirm that our failure to validate surface localization in certain strains was due to the protein being confined to the cytosol rather than simply failing to express. We confirmed the expression of a subset of these tagged proteins by western blotting for the HA tag. We detected robust expression of recA, hns, and secE proteins by western blotting at the correct molecular weights despite not detecting them by flow cytometry (Fig. S2). The hup protein fusion could not be detected by western blotting, suggesting that its overexpression is not well tolerated by H37Rv. Given these observations, we conclude that protease shaving as performed here must be combined with orthogonal validation to validate findings, as has been done for other compartments (26).

### Large protein fusions to PPE18 fail to export to the Mtb surface

**Fig 3:**
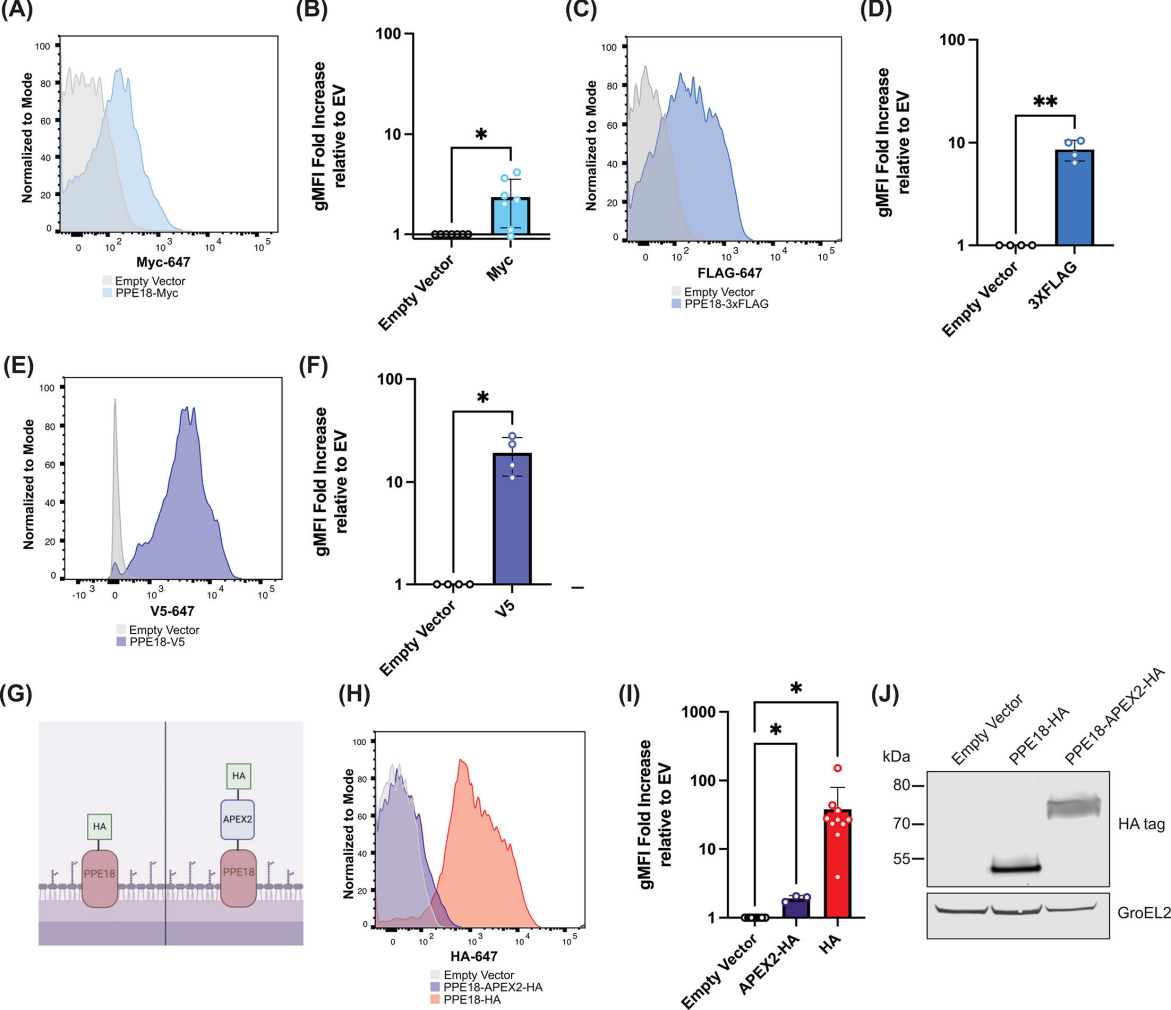
Small epitope tag fusions to PPE18, but not larger protein fusions, are robustly antibody accessible on Mtb. PPE18 fusions were made to Myc, 3×FLAG, and V5 and validated by flow cytometry. Quantification is shown relative to an empty vector (A and B: Myc; C and D: 3×FLAG; E and F: V5; *P < 0.05 and **P < 0.01; paired *t*-test, *n =* 4–7). (**G**) Diagram of PPE18-APEX2-HA fusion. (**H**) Flow cytometry analysis of HA expression on Mtb expressing PPE18-HA, PPE18-APEX2-HA, or an empty vector. (**I**) Relative quantification is normalized to the empty vector strain (*P < 0.05 and **P < 0.01; paired *t*-test, *n =* 3–8). (**J**) Western blot analysis of HA expression in proteins extracted from pellets of Mtb expressing PPE18-HA, PPE18-APEX2-HA, or an empty vector. An HA antibody was used to detect the tagged protein, and a GroEL2 antibody was utilized as a loading control.

We next sought to confirm that the observations made with PPE18 using an HA tagging strategy are generalizable to other epitope tags. We generated plasmids expressing PPE18 with C-terminal Myc (10 amino acids, 1.2 kDa), V5 (14 amino acids, 1.42 kDa), and 3×FLAG (24 amino acids, 2.73 kDa) tags. We subsequently transformed these plasmids into H37Rv. Indirect flow cytometry confirmed that all epitope tags tested are surface accessible (Fig. 3A through F, *n* = 4 independent biological replicates).

Given the success of adding epitope tags to PPE18, we next asked whether larger proteins could also be exported to Mtb’s surface. Anecdotally, the field has had variable and irreproducible success with this biological task. Several biotechnologies desired in the field would be enabled with an Mtb strain that can be functionalized with heterologous proteins, including surface display of heterologous enzymes to monitor bacterial infection, phagocyte interaction, and characterization of pathogen-specific compartments. Previous studies have shown that APEX2, a 28 kDa enzyme that facilitates proximity labeling, could be targeted to the periplasm of Mtb and *Mycobacterium smegmatis* (27, 28). We thus sought to determine if fusion of APEX2 containing a C-terminal HA tag to PPE18 (PPE18-APEX2-HA) would facilitate the export of APEX2 to the Mtb surface (Fig. 3G). Compared to an Mtb strain expressing PPE18-HA, we observed far less HA staining of the PPE18-APEX2-HA strain (Fig. 3H and I, *n* = 3–10 independent biological replicates). This could not be explained by the poor expression of the construct because western blot analysis of the HA-tagged protein revealed robust expression by Mtb (Fig. 3J, representative of three independent biological replicates). Similar conclusions were made using PPE18 with another proximity-labeling enzyme, TurboID (35 kDa) (Fig. S3, representative of three independent biological replicates). We next sought to understand whether this observation that there was a limited carrying capacity for PPE proteins. Similar experiments were performed with PPE51 where we attempted to fuse TurboID to the C terminus of PPE51 and PPE36, and we also did not detect any appreciable HA staining (Fig. S4, representative of three independent biological replicates). The epitope tags we tested ranged in size from 1.2 to 2.73 kDa, whereas the proteins we sought to fuse to PPE18 were larger (~30 kDa) leaving a large range of sizes for which we did not have information. We next extended the HA tag fused to PPE18 by ~1 kDa using the sequence scar that results from Gateway Cloning from the NIH BEI library of Mtb antigens (TQLSCTKW), and this resulted in a twofold decrease in HA staining, suggesting that the carrying capacity for additional protein biomass on this PPE protein is small (Fig. S5, *n* = 3 independent biological replicates).

### PPE18-ALFA tag protein fusions support the investigation of PPE18 localization and surface functionalization of Mtb

Given that PPE18 is an antigen included in the M72 vaccine, which is presently in vaccine trials, we sought to obtain an enhanced understanding of PPE18 localization on the Mtb surface using microscopy. While flow cytometry demonstrates the surface accessibility of PPE18, it cannot evaluate the spatial distribution of proteins along the bacterium (29).

**Fig 4:**
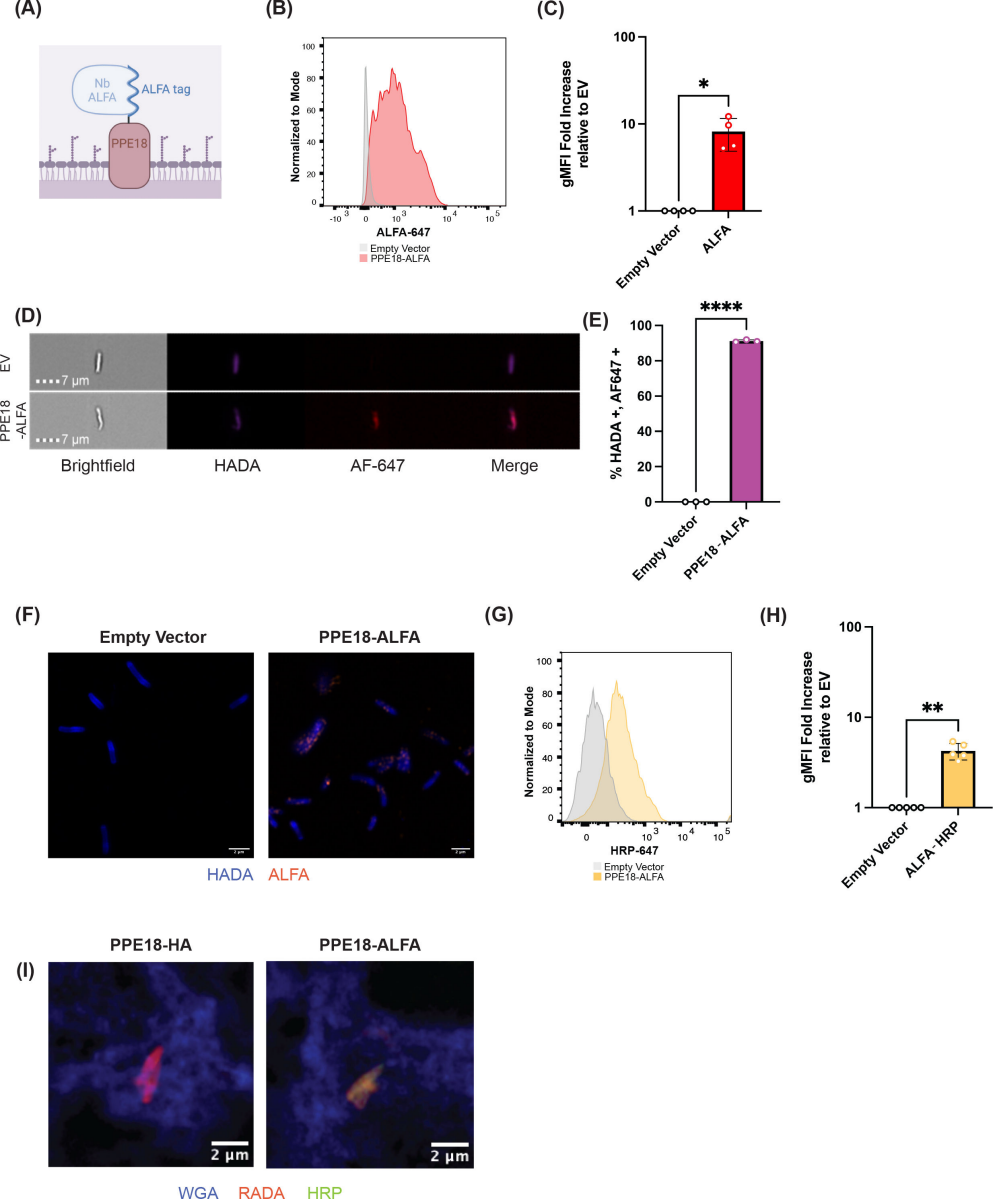
PPE18-ALFA fusions can be used to study protein localization and facilitate attachment of heterologous proteins to the Mtb surface. (**A**) Diagram of PPE18-ALFA fusion with bound anti-ALFA nanobody. (**B**) A PPE18 fusion was made to the ALFA tag, and flow cytometry was conducted using an anti-ALFA nanobody conjugated to AlexaFluor 647. Representative of four biological replicates. (**C**) Quantification of gMFI of ALFA signal across PPE18-ALFA and empty vector Mtb strains *(n =* 4, *P < 0.05, paired *t*-test). (**D**) Image cytometry analysis of anti-ALFA AlexaFluor 647 on HADA stained Mtb (empty vector [top] or PPE18-ALFA [bottom]). Brightfield, merge, and individual fluorescence channels are included as a composite image of four panels. Representative of three biological replicates. (**E**) Quantification of image cytometry analysis of anti-ALFA AlexaFluor 647 staining of Mtb expressing either an empty vector or PPE18-ALFA (****P < 0.0001, paired *t*-test). (**F**) Representative images of anti-ALFA staining of Mtb expressing either an empty vector or PPE18-ALFA using Airyscan microscopy (50×). Both Mtb strains were grown with HADA to visualize their cell walls (blue) and were stained for anti-ALFA AlexaFluor 647 (orange). Scale bar indicates 2 µm. Representative of three biological replicates. (**G**) Mtb strains expressing an empty vector or PPE18-ALFA were subject to surface conjugation with an anti-ALFA nanobody fusion to horseradish peroxidase (HRP). Following surface conjugation, indirect flow cytometry was performed to detect the presence of HRP on the Mtb surface. Representative of three biological replicates. (**H**) Quantification of gMFI of HRP signal across PPE18-ALFA and empty vector Mtb strains following conjugation to an anti-ALFA nanobody fusion to HRP (*n =* 3, **P < 0.01, paired *t*-test).

We generated an additional Mtb strain in which PPE18 was tagged with an ALFA tag (1.69 kDa), a rationally designed epitope tag for which a high-affinity nanobody reagent has been developed (30). Using flow cytometry, we first confirmed that the ALFA tag was surface accessible (Fig. 4A through C, *n* = 4 independent biological replicates). We utilized image cytometry to orthogonally confirm the spatial distribution of PPE18 on Mtb. Mtb expressing an empty vector or PPE18-ALFA were grown in the presence of HADA, a blue, fluorescent D-amino acid that labels Mtb cell wall peptidoglycans. Following HADA incorporation, cells were directly stained with an anti-ALFA nanobody conjugated to Alexa Fluor 647. We observed robust HADA incorporation in both empty vector and PPE18-ALFA strains. Among the cells that were HADA positive, the percentage of Mtb that were AF647 positive was significantly greater in the PPE18-ALFA strain than in the empty vector control (Fig. 4D and E, *n* = 3 independent biological replicates). The resolution of image cytometry was insufficient to determine if there are specific spatial distribution patterns of PPE18-ALFA on the Mtb surface, so we next turned to confocal microscopy. Imaging of the PPE18-ALFA strain grown axenically revealed a punctate distribution of PPE18 along the body of the bacterium, representing to our knowledge the first visual inspection of Mtb surface protein spatial distribution (Fig. 4F, representative of three independent biological replicates, Fig. S6). Similar findings were obtained when we imaged the PPE18-HA strain by confocal microscopy (Fig. S7, representative of three independent biological replicates). We furthermore validated that another Mtb outer membrane protein (PPE36) can be tagged with an ALFA tag and bind the anti-ALFA nanobody (Fig. S8).

Based on our success with the display of the ALFA tag on the Mtb surface and our lack of success in creating Mtb protein fusions to PPE18 that result in surface delivery of large heterologous proteins, we reasoned that an alternative strategy to leverage the surface proteome to create Mtb strains with novel functions on their surface would be to leverage the exquisite affinity of the anti-ALFA nanobody and fuse the nanobody to diverse proteins of interest. An approach like this is modular and can be improved upon as more affinity reagents are developed for native Mtb proteins and more epitope tags with companion affinity reagents are engineered. To demonstrate this principle, we utilized the PPE18-ALFA strain and functionalized the Mtb surface with horseradish peroxidase (HRP), an enzyme capable of performing proximity labeling reactions (31, 32). We grew Mtb expressing PPE18-ALFA or an empty vector and then incubated them with an anti-ALFA nanobody-HRP fusion. We next confirmed the conjugation of HRP to the Mtb surface using indirect flow cytometry. Compared to the empty vector Mtb strain, HRP was much more abundant on the PPE18-ALFA surface (Fig. 4G and H, *n* = 5 independent biological replicates). Taken together, these data demonstrate that a bipartite system using an Mtb strain engineered to express an epitope tag on the surface can be used as a chassis to deliver novel chemical moieties and proteins to the Mtb surface.

## DISCUSSION

Despite the importance of the mycobacterial surface to the regulation of Mtb physiology in axenic culture as well as in phagocytes, much of our understanding of the mycobacterial surface proteome remains incomplete. Prior studies in Mtb have identified surface proteins, but the techniques deployed to define these proteins focused on individual proteins and used laborious techniques such as ultracentrifugation to confirm their localization. Here, we build from approaches originally established in *Streptococcus pyogenes* to apply protease shaving to live, virulent Mtb. Using these approaches, we nominate and identify novel Mtb surface proteins, including an Mtb vaccine antigen, providing insight into the mechanism by which immunity following this vaccine might occur. We further demonstrate that defining this Mtb compartment proteome can be utilized to deliver heterologous proteins to the Mtb surface.

Several of the top proteins identified in protease shaving that were validated shared notable biological characteristics. Namely, many of these proteins belong to the PE and PPE family, known for their proline and glutamic acid residue-rich N-terminal sequences. We identified PE12, PE23, PPE10, PPE18, PPE20, PPE32, PPE33, PPE38, PPE40, PPE51, and PPE60 as having a fold change > 2 in our experiments. The PE/PPE proteins are hypothesized to have functions like nutrient transport, host-pathogen interaction, or immune evasion—functions that necessitate either their secretion or cell wall or surface localization (33). While the subcellular localization of PE/PPE family members remains incompletely defined, our findings suggest that many members are surface exposed. We saw both known surface proteins and expanded the list of proteins that are known to be surface accessible. For example, Su and colleagues (34) demonstrated that PPE60 was cell surface exposed through a recombinant BCG PPE60-His strain that underwent immunoblotting and a proteinase K degradation assay. Their study, along with another by Gong et al. (35), highlights the pro-inflammatory immunological role of PPE60, which includes the promotion of dendritic cell maturation and macrophage pyroptosis. We did not formally validate this protein in Mtb; however, it was the 11th ranked protein in our list. A previous study demonstrated that PPE38 is exposed on the surface of *Mycobacterium marinum*, and our results in Mtb demonstrate that this protein has a similar localization pattern (24, 25). PE/PPE proteins are thought to function as porins to facilitate nutrient transport. PPE20, which we were not able to validate by flow cytometry, has been implicated in calcium transport (36). Of the PPE proteins detected in our experiment, PPE38, PPE51, and PPE60 are among the best studied; however, only the molecules transported by PPE51 are known (14).

Of the 10 proteins tested for targeted follow up, PPE18 was of particular interest given its remarkable fold change by both flow cytometry and mass spectrometry. Although not previously shown to be on the Mtb surface, other studies have identified PPE18 by mass spectrometry in Triton X-114 extracts, cell membrane protein fraction, and whole cell lysates from Mtb H37Rv (37, 38). We demonstrated surface localization of this protein and found that it consistently had the highest fold change increase of geometric mean fluorescence intensity when compared to our empty vector control and all other proteins in this study. Moreover, we were able to show direct evidence of PPE18’s surface localization and spatial organization via microscopy. PPE18’s localization likely underscores its function. For instance, previous studies have suggested that PPE18 is a virulence factor that plays a role in IL-10 production by macrophages by interacting with TLR2 (34, 39). PPE18 is also one of the proteins included in an Mtb vaccine, M72/AS01E, and was originally chosen because of its ability to stimulate cell-mediated and antibody-mediated immune responses in both mice and controlled human infection (40). Specifically, PPE18 is one of a handful of PE/PPE proteins known to generate CD4-mediated T-cell responses, which was showcased in the high frequency of M72-specific CD4+ T cells after vaccination (41, 42). Additionally, antibodies are generated against PPE18 following vaccination (43). Antibodies against Mtb antigens have steadily gained research interest due to their ability to be elicited during vaccination, their ability to be used as diagnostics, and their role in mediating antimicrobial responses (44–49). Altogether, this further supports the ability of surface shaving to identify surface proteins of relevant clinical and therapeutic interest.

In addition to identifying surface proteins, we were able to discern several characteristics of surface functionalization for Mtb. One is the ability to localize small peptides, but not larger proteins, via fusion to surface-accessible Mtb proteins. Our ability to fuse five different epitope tags to PPE18 is in agreement with previous studies that have used similar-sized tags to detect the expression of recombinant proteins in mycobacteria. For larger proteins, previous strategies were devised that fuse signal peptides from validated substrates of the Sec protein secretion system in order to dictate the localization of an enzyme to a compartment of interest, like the periplasm (28). To anchor an enzyme of interest to the Mtb surface, we hypothesized that fusing it to a known surface protein might be one way to facilitate its export. We were not successful at achieving this goal, and several outstanding questions still need to be answered, including the upper limit of protein size to be fused, whether additional forms of optimization need to take place such as modulating protein expression level, and the underlying biological mechanisms of export to the Mtb surface. Nevertheless, our work provides a framework to display short binding motifs on the surface of Mtb.

Finally, we utilized our localization analysis in service of a biological problem that has not been successfully addressed in Mtb, the display of a heterologous protein on the Mtb surface. While other papers claim to have achieved this in Mtb, we were unable to reproduce any of these findings either due to lack of experimental detail in the methods or irreproducibility. We successfully leveraged the ALFA tag fused to PPE18 to functionalize the Mtb surface with a chemical moiety (AlexaFluor 647) and an enzyme, HRP. HRP is a peroxidase of interest because it can perform proximity labeling reactions; thus, our study lays the groundwork for future-specific study of the composition of the phagosomal lumen as has been done with other pathogens (50). For example, with a surface-localized HRP, we can potentially define macrophage surface proteins that Mtb interacts with. Akin to how immune cells modify their surface proteome during different states, our method can be applied to define if and how the Mtb surface proteome changes during stress conditions. For example, others have shown that PPE20 is downregulated following calcium exposure, so we reason it would be interesting to see if this transcriptional difference is also mirrored at the level of the surface proteome (36). We hypothesize that the Mtb surface proteome may dynamically change in response to environmental changes broadly, which may aid in elucidating the diverse functions of these PE and PPE proteins.

The approach we applied in this study is not without caveats. First, we optimized our biochemical workflow based on PPE51 as a prototype surface protein; however, what a prototypical Mtb surface protein is or whether one exists is not actually known. We used PPE51 because of the many previously published studies identifying Mtb surface proteins; this was the first one that worked after many failures. We hypothesize that future iterations of this assay can utilize all the surface proteins (PE and PPE proteins) we validated to further optimize surface shaving biochemical parameters. Furthermore, we utilized trypsin as our protease of interest in our assay, thus restricting ourselves only to identifying peptides from surface proteins that can be liberated using this enzyme. Given the modularity of our approach, other enzymes can be used to enrich our understanding of Mtb surface proteins. Additionally, our approach did not capture all rigorously validated known Mtb surface proteins, such as PPE36 and PPE62, among the top hits. This may reflect the inherent challenges in data-dependent mass spectrometry for discovery applications or may reflect the limited tryptic peptides accessible from these proteins on the Mtb surface. As with all mass spectrometry applications, to define the surface proteome, it is essential that the peptides we identify be uniquely attributable to a specific Mtb protein. This is inherently a challenge for the PE/PPE class of proteins where sequence repetition is common. Our validation strategy to make tagged strains is one of many potential approaches for validation. Alternative approaches that could have been used include ORBIT to tag the endogenous copy of the protein instead of overexpressing a tagged copy of the gene or raising a high-quality monoclonal antibody to the Mtb protein for flow cytometry.

We hypothesize that delivery of Mtb engineered to express proximity-labeling enzymes on the surface will facilitate future study of the composition of the Mtb-containing phagosome. Previous studies used mechanical homogenization paired with differential centrifugation or conjugation of magnetic beads onto Mtb’s surface to understand the composition (51, 52). Although several studies have started to characterize the Mtb phagosome with targeted methods, obtaining useful information about its dynamics, these methods focus on one-dimensional profiling of the pathogen-containing phagosome (53–56). Consequently, proximity labeling that is enabled by functionalization of the Mtb surface could facilitate the holistic, pure characterization of the lumenal contents of the Mtb-containing phagosome. One caveat about this proposed approach is that, unlike an engineered Mtb that expresses the proximity labeling enzyme on its own, surface-delivered enzyme will be in limited supply, potentially limiting the length of time this may be a useful tool in the proteolytic environment of the phagosome.

In conclusion, this work further characterizes the Mtb surface proteome and demonstrates that this compartment can be exploited to identify new vaccine targets and set the stage to enable biotechnologies for understanding pathogen interactions.

## MATERIALS AND METHODS

### Mtb culture for protease shaving and flow cytometry

Mtb H37Rv was grown in 7H9 media supplemented with 10% OADC (Thermofisher, B12351), 0.2% glycerol (Thermofishe, 17904), and 0.02% tyloxapol (VWR, TS42237-0050) to the mid-log phase (7H9-Tyloxapol).

### Plasmid construction and Mtb transformation

For the validation of protease shaving mass spectrometry results, full-length Mtb genes (without a C-terminal stop codon) were amplified from H37Rv genomic DNA. PCR amplicons were inserted by Gibson Assembly into a standard surface display vector consisting of a strong promoter (GroEL2), followed by a ScaI cut site for gene insertion, a glycine-serine linker, and an HA tag.

Additional PPE18 or PPE36 fusion strains (Myc, 3×FLAG, V5, ALFA, APEX2-HA, and TurboID-HA) were generated by Gibson assembly.

### Mtb culture for transformations and electroporation

Mtb H37Rv was grown in 7H9 media supplemented with 10% OADC (Thermofisher, B12351), 0.2% glycerol (Thermofisher, 17904), and 0.02% Tween-80 (Sigma-Aldrich, P1754-500ML) to the mid-log phase (7H9-OADC).

Wild-type H37Rv was grown in 7H9 to the mid-log phase. Bacteria were pelleted and washed with 10% glycerol three times and then resuspended in 400 µL of 10% glycerol per transformation. They were then transformed with 200 ng of plasmid via electroporation. Electroporated bacteria were recovered in 1 mL 7H9 for 48 hours. They were plated on 7H10 plates supplemented with 50 µg/mL hygromycin B (Millipore Sigma, 10843555001) for 17 days. Transformants were inoculated into 5 mL of 7H9 containing hygromycin B for 5 days prior to subsequent dilution and storage at −80°C.

### Mtb staining, surface conjugation, and flow cytometry

Mtb strains were grown in 7H9 with 50 µg/mL hygromycin B to an OD_600_ between 0.5 and 1. 1e8 bacteria from each strain were pelleted and resuspended in 500 µL of 4% paraformaldehyde (VWR, AAJ61899-AP) for 1 hour at room temperature. Following fixation, Mtb were pelleted for 5 minutes at 4,000 × *g* and washed with 500 µL FACS buffer (PBS without divalent cations, 2% heat-inactivated fetal bovine serum, and 2 mM EDTA). Bacteria were incubated with the indicated primary antibody (1:250), which included rabbit anti-HA (CST, C29F4 #3724), chicken anti-myc (exalpha, ACMYC), rabbit anti-3×FLAG (CST, D6W5B #14793), rabbit anti-V5 (CST, D3H8Q #13202) in FACS buffer for 30 minutes at room temperature. Alexa Fluor 647-conjugated secondary antibodies were added according to the species of the primary antibody (Thermofisher, A-21245, rabbit or Thermofisher, A-21449, chicken) at a 1:120 dilution for an additional 10 minutes in the dark. The bacteria were washed two times with FACS buffer, resuspended in 500 µL of FACS buffer, and analyzed by flow cytometry.

In the case of the ALFA tag, bacteria were stained directly with the FluoTag-X2 anti-ALFA AF647 (NanoTag, N1502) (1:500) for an hour, washed two times with FACS buffer, and resuspended in 500 µL FACS buffer prior to analysis by flow cytometry.

For surface conjugation of HRP to the Mtb surface, PPE18-HA- or PPE18-ALFA-expressing bacteria were stained with sdAB anti-ALFA HRP (NanoTag, N1505) (1:500 in FACS buffer) for an hour at room temperature. Bacteria were subsequently stained with an anti-HRP antibody (Thermofisher, MA1-10371) (1:250) in FACS buffer for 30 minutes at room temperature. Mtb were finally stained with an AF647-conjugated secondary antibody (1:120) for 10 minutes at room temperature prior to two washes with FACS buffer and analysis by flow cytometry.

### Protein shaving optimization experiments

Surface proteins of Mtb H37Rv were identified using a protocol adapted from prior literature (20). A volume of 10 mL of H37Rv-expressing PPE51-HA was grown in 7H9-Tyloxapol to the mid-log phase. 1e8 bacteria from each replicate per condition were pelleted at 3,000 × *g* for 5 minutes, washed once in PBS without divalent cations (Corning, 21-040-CV), and resuspended in a 100 µL 40% sucrose/PBS buffer. For experimental conditions where Mtb were exposed to protease, either 0.4 or 0.05 µg/mL of trypsin (Promega, V5113) was added to the sucrose buffer. For the negative control condition, PBS without divalent cations was added. Samples with protease were incubated for 10, 30, or 60 minutes. Following incubation, all samples were then pelleted at 3,000 × *g* for 5 minutes, washed once in PBS, and resuspended in 500 µL of 4% paraformaldehyde for 1 hour at room temperature. Samples were then stained for flow cytometry as described above.

### Protein surface shaving protocol

A volume of 100 mL of Mtb WT H37Rv was grown in 7H9-Tyloxapol per replicate to the mid-log phase. Bacteria were pelleted by centrifugation at 3,000 × *g* for 10 minutes at 4°C and resuspended in PBS. A total of three washes were performed. Following the third wash, the Mtb pellet was resuspended in a 40% sucrose/PBS buffer at a ratio of 100 µL of buffer for every 100 mL of bacterial culture. For the protease shaving experimental conditions, 0.4 µg/mL of trypsin was added, and for control conditions, PBS was added. The suspension was incubated for 30 minutes at 37°C. Samples were pelleted by centrifugation at 3,000 × *g* for 10 minutes at 4°C, and the supernatant was recovered. The supernatant was filtered with a 0.2 µm pore-size filter (Pall, ODM02C35) twice. For the autolysis control conditions, 0.4 µg/mL was added to the cell-free supernatant from pelleted bacteria from parallel preparations from the surface shaving experiment and incubated for 30 minutes at 37°C. Oasis HLB Extraction Cartridges (Waters, WAT094225) were used to clean the supernatants for mass spectrometry preparation. The cartridges were equilibrated with 0.6 mL of 80% acetonitrile (Sigma-Aldrich, 34851-1L) and subsequently 0.6 mL of 0.1% formic acid (Sigma-Aldrich, 33015). Samples were loaded at a ratio of 300 µL of supernatant to 300 µL of PBS. Each sample was washed twice with 0.6 mL of 2% acetonitrile/0.1% formic acid. Elution of the samples was done in three steps with 0.6 mL of the following solutions: 10% acetonitrile/0.1% formic acid, 20% acetonitrile/0.1% formic acid, and 50% acetonitrile/0.1% formic acid, each collected in its own tube. All samples were snap-frozen in liquid nitrogen and lyophilized.

### Tandem mass tag labeling

Peptides for mass spectrometry were prepared for tandem mass tag (TMT) analysis according to the manufacturer’s instructions (Thermofisher, 90061). The tryptic peptides were resuspended in 100 µL of 100 mM TEAB, then vortexed and briefly centrifuged. Forty-one microliters of anhydrous acetonitrile was added to each of the TMT label reagents. They were vortexed and briefly centrifuged. The 41 µL of the TMT reagents was added to 100 µL of sample, vortexed, and briefly centrifuged. The samples were incubated for 1 hour at room temperature. After an hour, 8 µL of 5% hydroxylamine was added to each sample and incubated for 15 minutes to quench the reaction. Equal amounts of each sample were then combined and placed into a speed-vac to dry completely.

### LC-MS-MS and quantitative analysis

The TMT-labeled tryptic peptides were resuspended in 10 µL of 0.2% formic acid, and 1 µL was injected. The tryptic peptides were separated by reverse phase HPLC (Thermofisher Ultimate 3000) using a Thermo PepMap RSLC C18 column (2 µm tip, 75 µm × 50 cm PN# ES903) over a gradient before nano-electrospray using a ThermoFisher Orbitrap Exploris 480 mass spectrometer. Solvent A was 0.1% formic acid in water, and solvent B was 0.1% formic acid in acetonitrile. The gradient conditions were 1% B (0–10 minutes at 300 nL/minute), 1% B (10–15 minutes, 300–200 nL/minute), 1%–10% B (15–20 minutes, 200 nL/minute), 10%–25% B (20–65 minutes, 200 nL/minute), 25%–36% B (65–75 minutes, 200 nL/minute), 36%–80% B (75–75.5 minutes, 200 nL/minute), 80% B (75.5–80 minutes, 200 nL/minute), 80%–1% B (80–80.1 minutes, 200 nL/minute), and 1% B (80.1–100 minutes, 200 nL/minute).

The mass spectrometer was operated in a data-dependent mode. The parameters for the full scan MS were a resolution of 120,000 across 375–1,600 *m/z* and a maximum IT of 25 ms. The full MS scan was followed by MS/MS for as many precursor ions in a 2 second cycle, with a NCE of 36, dynamic exclusion of 30 s, and resolution of 30,000.

Raw mass spectral data files (.raw) were searched using Sequest HT in Proteome Discoverer (Thermofisher). Sequest search parameters were 10 ppm mass tolerance for precursor ions; 0.05 Da for fragment ion mass tolerance; two missed cleavages of trypsin; fixed modifications were carbamidomethylation of cysteine and TMT modification on the lysines and peptide N-termini; variable modifications were methionine oxidation, methionine loss at the N-terminus of the protein, acetylation of the N-terminus of the protein, and also Met-loss plus acetylation of the protein N-terminus.

Data were searched against a *Mycobacterium tuberculosis* database downloaded from UniProt and a contaminant database made in-house at the Koch Institute Proteomics Core. The search criteria were as follows: FDR for peptide matches of high confidence was set at 0.01, Sequest HT Xcorr ≥ 2.00, and maximum delta mass was set at 3 PPM.

The statistical significance of a protein’s fold change was calculated by performing paired *t* tests. First, for each replicate, the experimental and control condition abundance levels were divided by the corresponding replicate’s control abundance level. These ratio groups were the inputs to Python’s scipy.stats.ttest_rel function. Two-sided hypothesis and *P*-values were obtained. The *P*-values were then adjusted using Python’s statsmodels.stats.multitest.fdrcorrection function for the Benjamini/Hochberg correction. In the cases where the control abundance levels were zero but nonzero in the experimental conditions, values were imputed to be 500—the lowest raw abundance levels seen, to obtain a fold change ratio. To be considered a potential surface protein, a candidate protein had to have at least two peptides obtained from protease cleavage, greater than twofold log change between the experimental (live, intact Mtb) versus control (cell-free supernatant), and have an adjusted *P*-value of less than 0.1. As all top proteins would be experimentally validated via epitope tagging, the adjusted *P*-value was chosen as 0.1 to balance the likelihood of the type I error occurring by exploring the search space for potential surface proteins.

### Western blotting

Mtb were first grown in 7H9-Tyloxapol to an OD_600_ of 0.8–1. Bacteria were pelleted and lysed in RIPA buffer (VWR, 97063-270) with 1× Halt Protease Inhibitors (Thermofisher, 78430). Bead beating was performed with Lysing Matrix B (MP Biomedical, 16911050-CF) for three rounds of agitation, 45 s each. The culture supernatants were filtered twice through a 0.2 µm filter (Pall, ODM02C35). Protein concentration was determined by BCA assay (Thermofisher), and equal amounts of protein lysate were denatured and prepared as recommended by the manufacturer for western blotting with Bolt 4%–12% Bis-Tris gels (Thermofisher, NW04120BOX). Gels were transferred to nitrocellulose membranes using an iBlot 2 (Thermofisher, 25-107). Membranes were blocked with Odyssey Blocking Buffer (Licor, 927-60003) and stained with the indicated primary antibodies. They were then stained with secondary antibodies, washed, and imaged on the Licor Odyssey imaging system.

### Immunofluorescence microscopy of axenic culture

Mtb cultures of the empty vector strain or PPE18-ALFA strain were grown to the mid-log phase in the presence of HADA (BioTechne, 6647/5) (1:500). 1e8 bacteria from each strain were incubated with anti-ALFA AF647 (Nanotag, N1502) (1:500), washed twice with PBS, and resuspended in 500 µL of 4% paraformaldehyde for 1 hour at room temperature. After fixation, both strains were washed once in PBS, and 100 µL of each strain resuspended in PBS was pipetted onto a 35 mm dish (Mattek). After evaporation of excess PBS, the strains were covered with a 1% agarose pad. Strains were imaged using a Zeiss CellDiscoverer 7 Microscope at a 50× objective lens.

Mtb cultures of the empty vector strain or PPE18-HA strain were grown to the mid-log phase in the presence of HADA (BioTechne, 6647/5) (1:500). 1e8 bacteria from each strain were resuspended in 500 µL of 4% paraformaldehyde for 1 hour at room temperature. After washing once with PBS, strains were stained with the rabbit anti-HA primary antibody (CST, C29F4 #3724) (1:250) in FACS buffer for 30 minutes at room temperature. Fluorescent AF647-conjugated goat anti-rabbit (Thermofisher, A-21245) secondary antibody (1:120) was added and incubated for another 10 minutes in the dark. The bacteria were washed two times with FACS buffer, and 100 µL of each strain resuspended in FACS buffer was pipetted onto a 35 mm dish (Mattek, P35G-1.5–14-C). After evaporation of excess FACS, the strains were covered with a 1% agarose pad. Strains were imaged at 100× magnification using a Zeiss CellDiscoverer 7 Microscope with a 50× objective and 2× tube lens.

Image cytometry was performed using a Cytek ImageStream. Brightfield, AF647, and HADA channels were collected. Images were analyzed using ImageStream software, and images were exported using the ImageStream software, which provides a composite image in addition to individual channels (four images per cell).

### Statistical analyses

Unless otherwise indicated, all statistical tests were performed in GraphPad Prism 10.

## Data Availability

All the data supporting this work are included in the figures or can be found in the supplementary information. Mass spectrometry data have been uploaded to the PRIDE Database under accession PXD052936. Plasmids for PPE18-HA and PPE18-ALFA have been deposited to Addgene.

## SUPPLEMENTAL MATERIAL

Supplemental figures spectrum.02277-24-s0001.pdf Fig. S1 to S8.

Supplemental material spectrum.02277-24-s0002.docx Separate supplemental table legends.

Table S1 spectrum.02277-24-s0003.xlsx Unlabeled mass spec data.

Table S2 spectrum.02277-24-s0004.xlsx TMT mass spec data.

ASM does not own the copyrights to Supplemental Material that may be linked to, or accessed through, an article. The authors have granted ASM a non-exclusive, world-wide license to publish the Supplemental Material files. Please contact the corresponding author directly for reuse.
