# Supplementary material for: Protease shaving of Mycobacterium tuberculosis facilitates vaccine antigen discovery and delivery of novel cargoes to the Mtb surface
Source: Microbiol Spectr. 2024 Dec 17;13(2):e02277-24. doi: 10.1128/spectrum.02277-24 (PMC11792546; doi:10.1128/spectrum.02277-24)
Supplement: Supplemental figures — Fig. S1 to S8. [file spectrum.02277-24-s0001.pdf]

Supplemental Figure 1

>Mycobacterium tuberculosis H37Rv|Rv1387|PPE20|9% coverage

MTEPWIAFPPEVHSAMLNYGAGVGPMLISATQNGELSAQYAEAASEVEELLGVVASEG
WQGQAAEAFVAAYMPFLAWLIQASADCVEMAAQQHVVIEAYTAAVELMPTQVELAANQ
IKLAVLVATNFFGINTIPIAINEAEYVEMWVRAATTMATYSTVSRSALSAMPHTSPPPLILK
SDELLPDTGEDSDEDGHNHGGHSHGGHARMIDNFFAEILRGVSAGRIVWDPVNGTLN
GLDYDDYVYPGHAIWWLARGLEFFQDGEQFGELLFTNPTGAFQFLLYVVVVDLPTHIA
QIATWLGQYPQLLSAALTGVIAHLGAITGLAGLSGLSAIPSAAIPAVVPELTPVAAAPPML
AVAGVGPAVAAPGMLPASAPAPAAAAGATAAGPTPPATGFGGFPPYLVGGGGPGIGFG
SGQSAHAKAAASDSAAAESAAQASARAQARAARRGRSAAKARGHRDEFVTMDMGF
DAAAPAPEHQPGARASDCGAGPIGFAGTVRKEAVVKAAGLTTLAGDDFGGGPTMPMM
PGTWTHDQGVFDEHR

>Mycobacterium tuberculosis H37Rv|Rv2737c|recA|11% coverage

MTQTPDREKALELAVAQIEKSYGKGSVMRLGDEARQPISVIPTGSIALDVALGIGGLPRG
RVIEIYGPESSGKTTVALHAVANAQAAGGVAAFIDAEHALDPDYAKKLGVDTDSLLVSQP
DTGEQALEIADMLIRSGALDIVVIDSVAALVPRAELEGEMGDSHVGLQARLMSQALRKM
TGALNNSGTTAIFINQLRDKIGVMFGSPETTTGGKALKFYASVRMDVRRVETLKDGTNA
VGNRTRVKVVKNKCLAEGTRIFDPVTGTTHRIEDVVDGRKPIHVVAAAKDGTLHARPVV
SWFDQGTRDVIGLRIAGGAIVWATPDHKVLTEYGWRAAGELRKGDRVAQPRRFDGFG
DSAPIPADHARLLGYLIGDGRDGWVGGKTPINFINVQRALIDDVTRIAATLGCAAHPQG
RISLAIAHRPGERNGVADLCQQAGIYGKLAWEKTIPNWFFEPDIAADIVGNLLFGLFESD
GWVSREQTGALRVGYTTTSEQLAHQIHWLLLRFGVGSTVRDYDPTQKRPSIVNGRRIQ
SKRQVFEVRISGMDNVTAFAESVPMWGPRGAALIQAIPEATQGRRRGSQATYLAAEMT
DAVLNYLDERGVTAQEAAAMIGVASGDPRGGMKQVLGASRLRRDRVQALADALDDKF
LHDMLAEELRYSVIREVLPTRRARTFDLEVEELHTLVAEGVVVHNCSPPFKQAEFDILYG
KGISREGSLIDMGVDQGLIRKSGAWFTYEGEQLGQGKENARNFLVENADVADEIEKKIK
EKLGIGAVVTDDPSNDGVLPAPVDF

>Mycobacterium tuberculosis H37Rv|Rv2352c|PPE38|9% coverage

**MILDFSWLPPEINSARIYAGAGSGPLFMAAAAWEGLAADLRASASSFDAVIAGLAAG
PWSGPASVAMAGAAAPYVGWLSAAAGQAELSAGQATAAATAFEAALAATVHPAAVT
ANRVLLGALVATNILGQNTPAIAATEFDYVEMWAQDVGAMVGYHAGAAAVAETLTPF
SVPPLDLAGLASQAGAQLTGMATSVSAALSPIAEGAVEGVPAVVAAAQSVAAGLPVD
AALQVGQAAAYPASMLIGPMMQLAQMGTTANTAGLAGAEAAGLAAADVPTFAGDIA
SGTGLGGAGGLGAGMSAELGKARLVGAMSVPPTWEGSVPARMASSAMAGLGAMP
AEVPAAGGPMGMMPMPMGMGGAGAGMPAGMMGRGGANPHVVQARPSVVPRVGI
G**

>Mycobacterium tuberculosis H37Rv|Rv0638|secE1|33% coverage

VSDEGDVADEAVADGAENADSRGSGGRTALVTKPVVRPQRPTGKRSRSRAAGADAD
VDVEEPSTAASEATGVAKDDSTTKAVSKAARAKKASKPKARSVNPIAFVYNYLKQVVAE
MRKVIWPNRKQMLTYTSVVLAFLAFMVALVAGADLGLTKLVMLVFG

>Mycobacterium tuberculosis H37Rv|Rv2986c|hupB|31% coverage

MNKAELIDVLTQKLGSDRRQATAAVENVVDTIVRAVHKGDSVTITGFGVFEQRRRAARV
ARNPRTGETVKVKPTSVPAFRPGAQFKAVVSGAQRLPAEGPAVKRGVGASAAKKVAK
KAPAKKATKAAKKAATKAPARKAATKAPAKKAATKAPAKKAVKATKSPAKKVTKAVKKTA
VKASVRKAATKAPAKKAAAKRPATKAPAKKATARRGRK

>Mycobacterium tuberculosis H37Rv|Rv3852|hns|30% coverage

MPDPQDRPDSEPSDASTPPAKKLPAKKAAKKAPARKTPAKKAPAKKTPAKGAKSAPPK
PAEAPVSLQQRIETNGQLAAAAKDAAAQAKSTVEGANDALARNASVPAPSHSPVPLIVA
VTLSLLALLLIRQLRRR

>Mycobacterium tuberculosis H37Rv|Rv2328|PE23|7% coverage

**MQFLSVIPEQVESAAQDLAGIRSALSASYAAAAGPTTAVVSAAEDEVSTAIASIFGAYG
RQCQVLSAQASAFHDEFVNLLKTGATAYRNTEFANAQSNVLNAVNAPARSLLGHPSA
AESVQNSAPTLGGGHSTVTAGLAAQAGRAVATVEQQAAAAVAPLPSAGAGLAQVVN
GVVTAGQGSAAKLATALQSAAPWLAKSGGEFIVAGQSALTGVALLQPAVVGVVQAG
GTFLTAGTSAATGLGLLTLAGVEFSQGVGNLALASGTAATGLGLLGSAGVQLFSPAF
LLAVPTALGGVGSLAIAVVQLVQGVQHLSLVVPNVVAGIAALQTAGAQFAQGVNHTML
AAQLGAPGIAVLQTAGGHFAQGIGHLTTAGNAAVTVLIS**

>Mycobacterium tuberculosis H37Rv|Rv1196|PPE18|45% coverage

**MVDFGALPPEINSARMYAGPGSASLVAAAQMWDSVASDLFSAASAFQSVVWGLTVG
SWIGSSAGLMVAAASPYVAWMSVTAGQAELTAAQVRVAAAAYETAYGLTVPPPVIAE
NRAELMILIATNLLGQNTPAIAVNEAEYGEMWAQDAAAMFGYAAATATATATLLPFEE
APEMTSAGGLLEQAAAVEEASDTAAANQLMNNVPQALQQLAQPTQGTTPSSKLGG
LWKTVSPHRSPISNMVSMANNHMSMTNSGVSMTNTLSSMLKGFAPAAAAQAVQTAA
QNGVRAMSSLGSSLGSSGLGGGVAANLGRAASVGSLSVPQAWAAANQAVTPAARA
LPLTSLTSAAERGPGQMLGGLPVGQMGARAGGGLSGVLRVPPRPYVMPHSPAAG**

Supplemental Figure 2

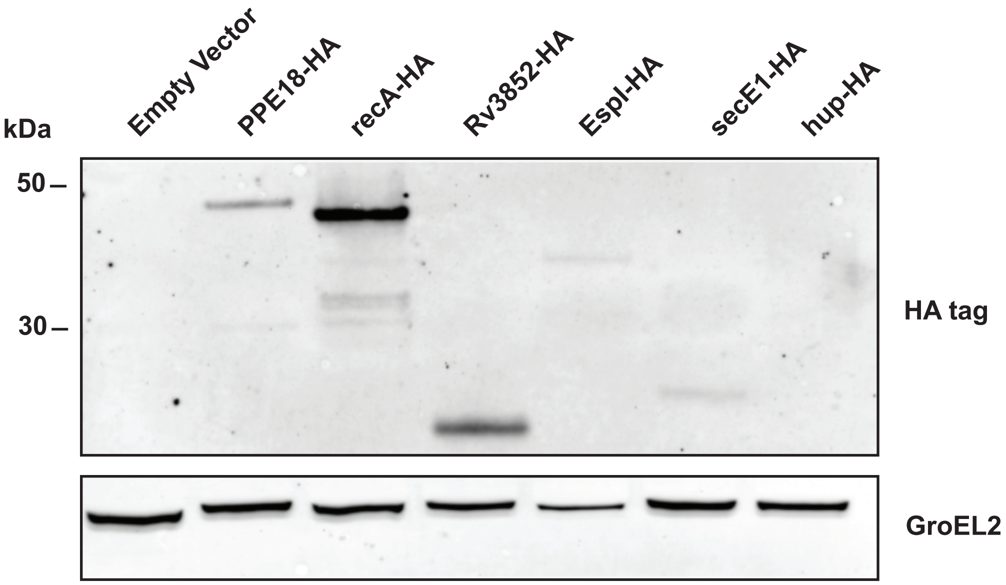

Supplemental Figure 3

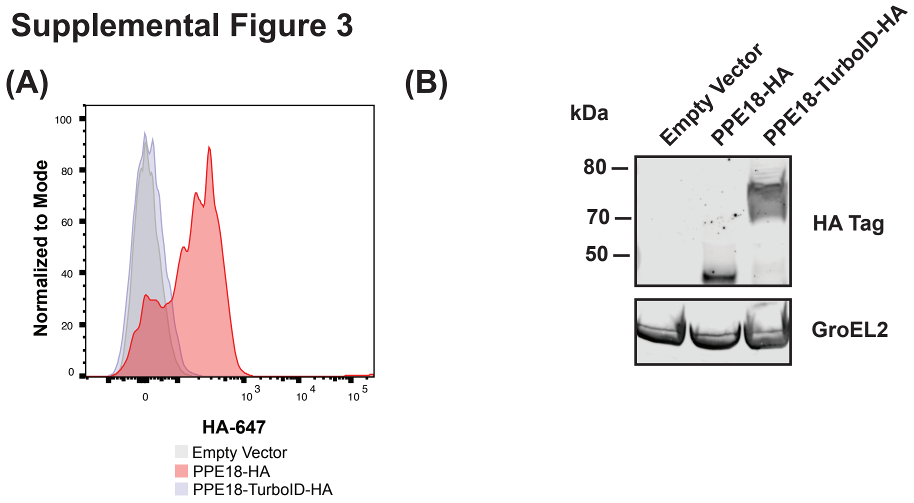

Supplemental Figure 4

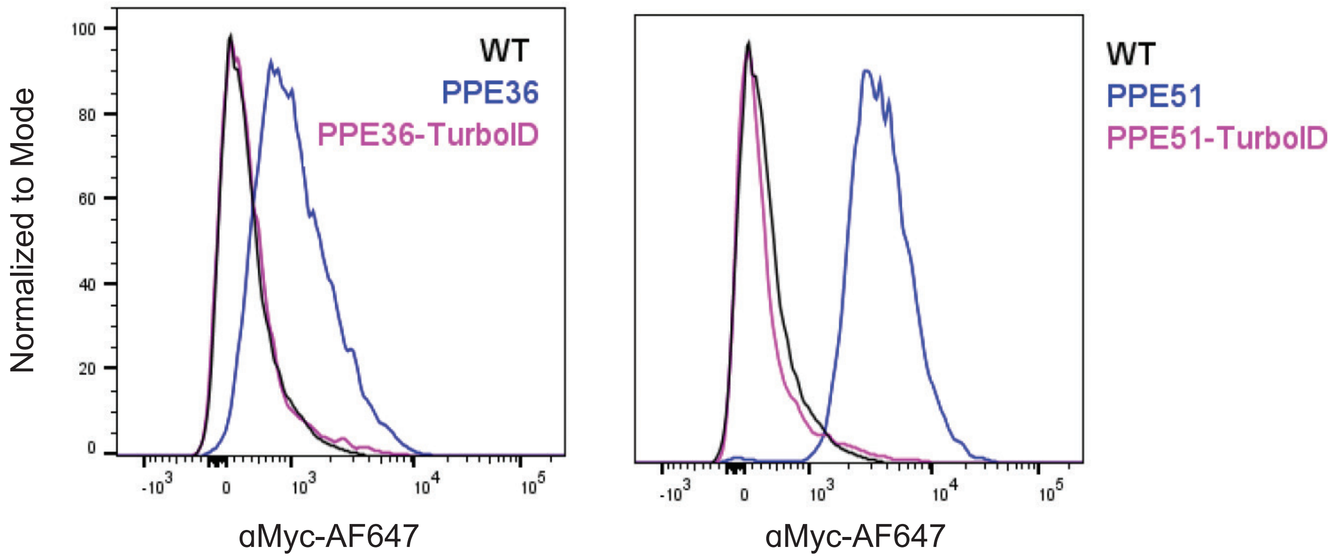

Supplemental Figure 5

(A)

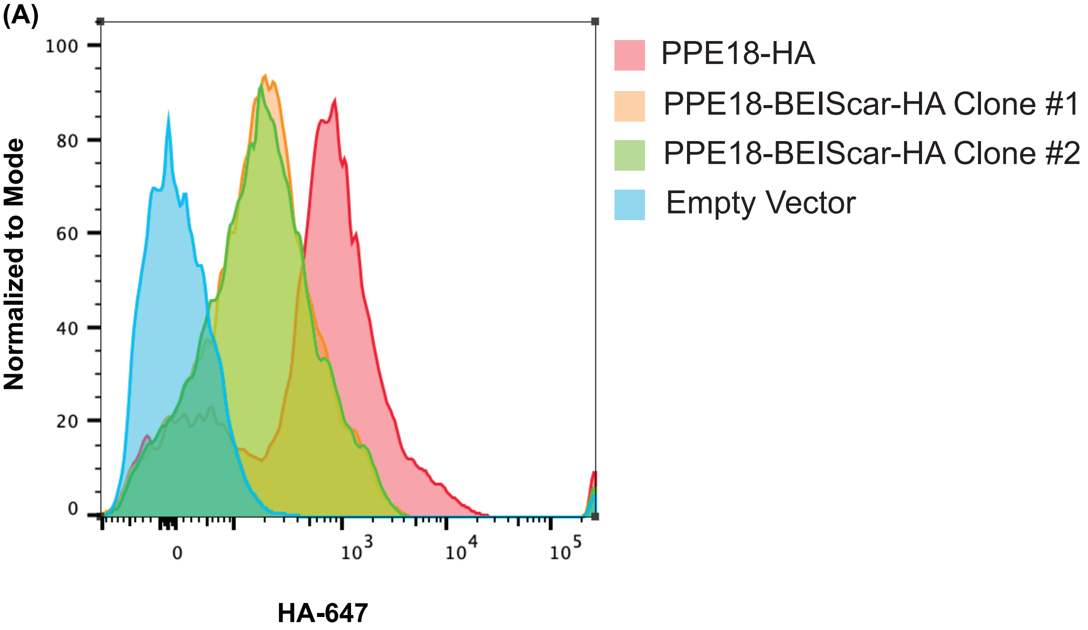

(B)

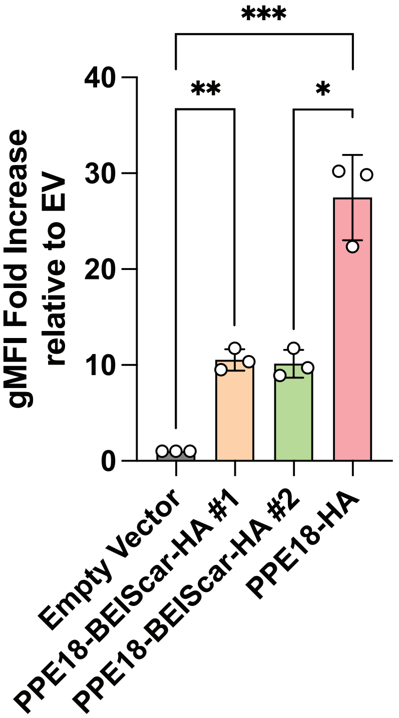

Supplemental Figure 6

Empty Vector

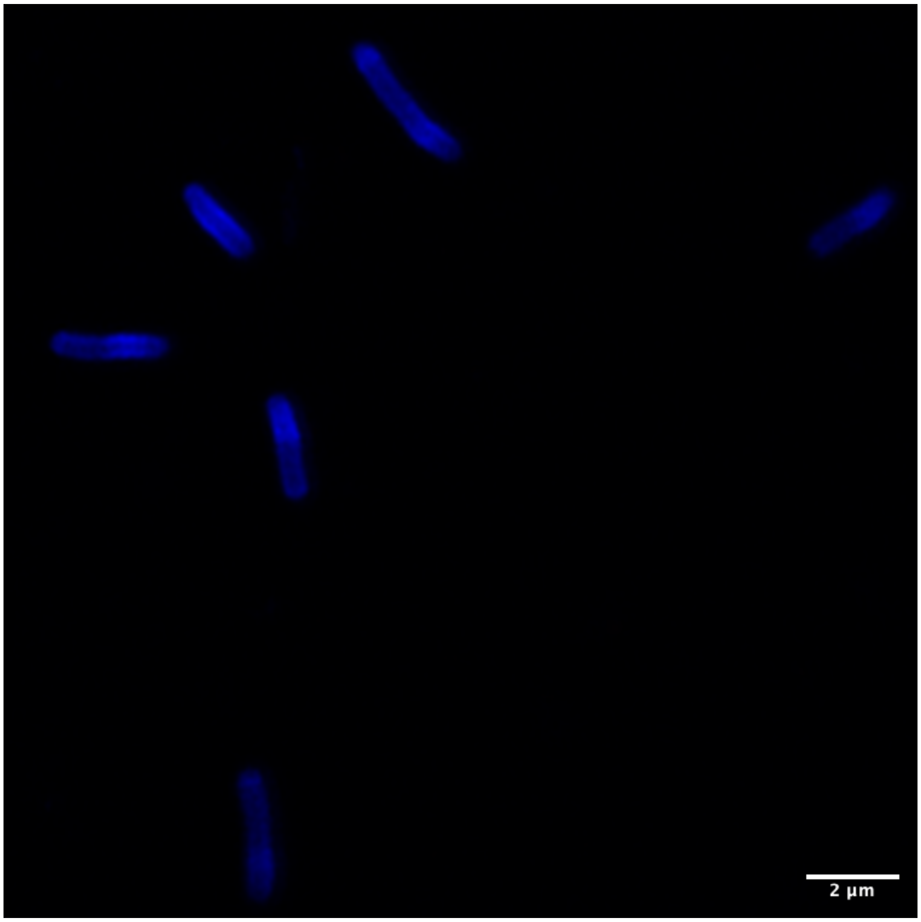

PPE18-ALFA

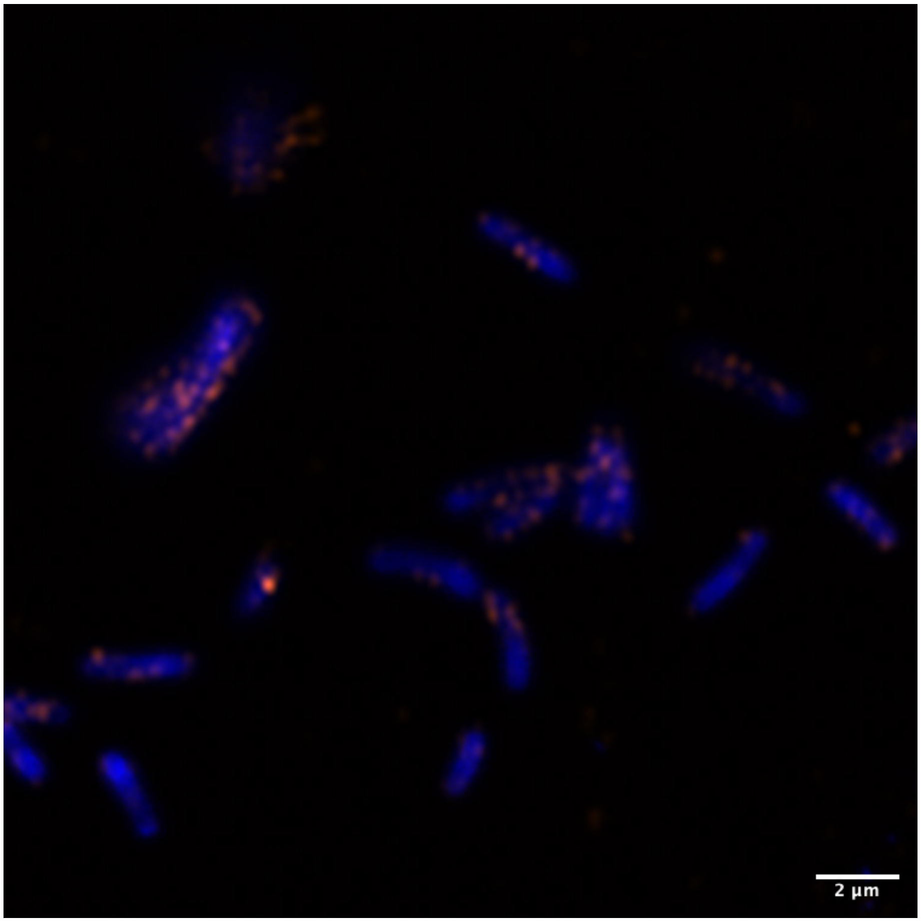

HADA HA

Supplemental Figure 7

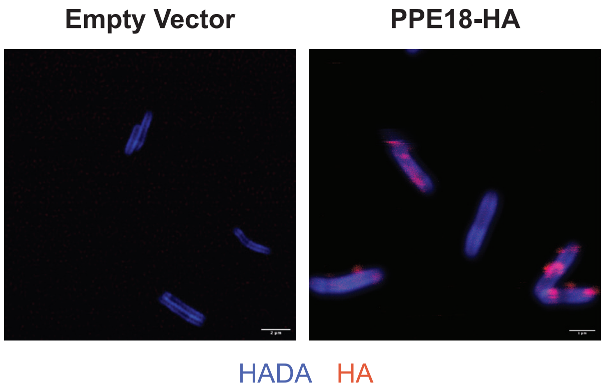

Supplemental Figure 8

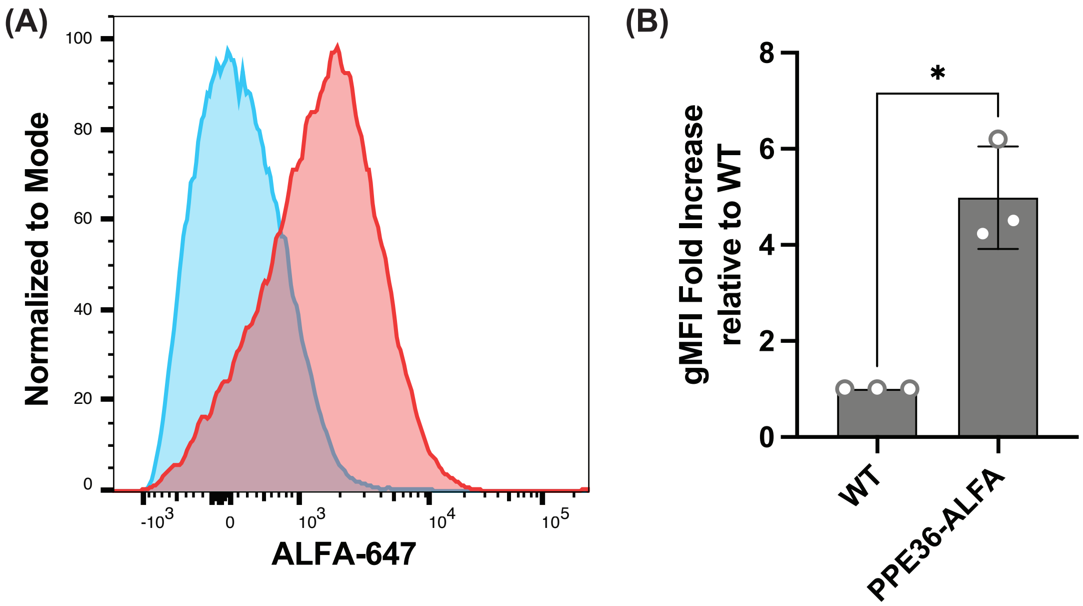

Supplemental Figure Captions

Supplemental Figure 1: Analysis of peptides identified from Mtb proteins for which epitope tagged validation strains were constructed. The full sequence of each Mtb protein is presented. Red text indicates that this peptide was identified by mass spectrometry. Black indicates it was not. Bold sequences validated in the surface shaving experiments.

Supplemental Figure 2: Western blot analysis of HA protein fusions to Mtb proteins identified by protease shaving. Mtb expressing an empty vector, PPE18-HA, recA-HA, Rv3852-HA, espI-HA, secE1-HA, and hup-HA were grown to mid-log phase, pelleted, and lysed. Bacterial lysates were probed for the presence of the HA epitope tag and for the GroEL2 protein as a loading control.

Supplemental Figure 3: HA signal on PPE18-TurboID-HA strains. (A) Flow cytometry analysis of HA signal on Mtb expressing PPE18-HA, PPE18-TurboID-HA or empty vector stained with an anti-HA antibody. (B) Western blot analysis of HA expression in proteins extracted from pellets of Mtb expressing PPE18-HA, PPE18-TurboID-HA or an empty vector. An HA antibody was used to detect the tagged protein and a GroEL2 antibody was utilized as a loading control.

Supplemental Figure 4: HA signal on PPE36 and PPE51 fusion strains. (A) Flow cytometry analysis of HA signal on Mtb expressing PPE36-HA, PPE36-TurboID-HA or WT Mtb stained with an anti-HA antibody.

Supplemental Figure 5: Flow cytometry analysis of linker extension on PPE18-HA strains. (A) Flow cytometry analysis of HA signal on Mtb expressing PPE18-HA, PPE18-BEIScar-HA or empty vector Mtb stained with an anti-HA antibody. (B) Quantification of gMFI of HA signal across Mtb strains (* $p<0.05$, ** $p< 0.005$, paired t-test, $n = 3$).

Supplemental Figure 6: Microscopy analysis of ALFA localization on Mtb expressing an empty vector or PPE18-HA. Representative images of ALFA localization on Mtb expressing PPE18-ALFA or an empty vector Mtb strains. Images were obtained using Airyscan microscopy (100x). HADA was used to visualize Mtb cell walls. Scale bar indicates 1 µm for PPE18-HA and 2 µm for the empty vector.

Supplemental Figure 7: Microscopy analysis of HA localization on Mtb expressing an empty vector or PPE18-HA. Representative images of HA localization on Mtb expressing PPE18-HA or an empty vector Mtb strains. Images were obtained using Airyscan microscopy (100x). HADA was used to visualize Mtb cell walls. Scale bar indicates 1 µm for PPE18-HA and 2 µm for the empty vector.

Supplemental Figure 8: PPE36 can accommodate tagging with an ALFA tag. (A) Flow cytometry analysis of ALFA signal on WT Mtb or Mtb expressing PPE36-ALFA stained with an anti-ALFA nanobody. (B) Quantification of gMFI of ALFA signal across Mtb strains (* $p<0.05$, paired t-test, $n = 3$).
