# Supplementary material for: Protease shaving of Mycobacterium tuberculosis facilitates vaccine antigen discovery and delivery of novel cargoes to the Mtb surface
Source: Microbiol Spectr. 2024 Dec 17;13(2):e02277-24. doi: 10.1128/spectrum.02277-24 (PMC11792546; doi:10.1128/spectrum.02277-24)
Supplement: Supplemental material — Separate supplemental table legends. [file spectrum.02277-24-s0002.docx]

**Supplemental Table 1: Results from unlabeled mass spectrometry surface shaving experiment with 0.4 μg/mL trypsin.** Mass spectrometry data summary.

**Supplemental Table 2: Results from protease shaving with 0.4 μg/mL trypsin.** Quantification of all Mtb proteins detected in mass spectrometry experiments.
